# Data Integration Innovations to Enhance Analytic Utility of Clinical Trial Content to Inform Health Disparities Research

**DOI:** 10.3389/fonc.2018.00365

**Published:** 2018-09-11

**Authors:** Steven B. Cohen, Jennifer Unangst

**Affiliations:** Division for Statistical and Data Sciences, RTI International, Washington, DC, United States

**Keywords:** project data sphere, data integration, MEPS, clinical trials, health disparities

## Abstract

Project Data Sphere (PDS) is a research platform that provides the research community with broad access to both de-identified patient-level data from oncology clinical trials and related analytic tools. While these data are rich in measures that characterize the clinical trials under study, data providers are required to de-identify patient-level data by removing key demographic data. To address these analytic constraints, the data profiles in selected PDS patient-level cancer phase III clinical datasets have been augmented by linking the social, economic, and health-related characteristics of like cancer survivors from nationally representative health and health care-related survey data. Using statistical linkage and model-based techniques, patient-level records in selected PDS datasets have been linked to those of comparable cancer survivors, and are thereby augmented with survey content on social, economic, and health-related characteristics. These new analytically enhanced PDS data resources enable more targeted analyses designed to examine questions such as how disparities in cancer patients' access to health care and income impact patient outcomes in specific phase III clinical trials, and what variations in patient outcomes are associated with specific demographic, socioeconomic, and health-related factors. This study provides an overview of the methodologies used to connect patient-level clinical trial data with nationally representative health-related data on cancer survivors from the national Medical Expenditure Panel Survey (MEPS). MEPS was designed to provide national population-based health care use, expenditure, and source of payment estimates in addition to measures of health status, demographic characteristics, employment, health insurance coverage, and access to health care. Study findings include probabilistic assessments of the representation of the patients in the respective clinical trials relative to the characteristics of cancer survivors in the general population. The study also demonstrates how the augmented datasets serve to enable researchers to assess the impact of socioeconomic factors added through data integration on cancer survival and related outcomes of interest.

## Introduction

Cancer researchers continue to advance discoveries and treatment protocols, yet every year, millions of lives are lost to cancer. Solutions are not advancing quickly enough. Researchers work independently and must often compete for resources needed to carry out their work. Project Data Sphere, LLC (PDS) was formed in 2012 to catalyze cancer research by bringing together diverse minds and technologies to help unleash the full potential of existing clinical trial data. PDS, an independent initiative of the CEO Roundtable on Cancer's (CEORT's) Life Sciences Consortium, operates a first-of-its-kind research platform. PDS provides the research community with broad access to both de-identified patient-level data from oncology clinical trials and freely available analytic tools to assist them in analyzing those data.

A primary goal of PDS is to advance new research efforts that will improve the lives of cancer patients and their families around the world (1, 2). These data are rich in measures that characterize the clinical trials under study, treatment protocols, and patient outcomes. However, to address the confidentiality provisions inherent to the trials, data providers are required to de-identify patient-level data prior to uploading datasets to ProjectDataSphere.org by masking or removing certain demographic data. Consequently, the influence of health-related and socioeconomic factors, access to and use of health care services, and predisposition of health behaviors on treatment effects and patient outcomes cannot currently be assessed. The inclusion of these measures would significantly enhance the analytic capacity and utility of the PDS data, further stimulating hypothesis generation and the initiation of new studies that explore these relationships.

The primary analytic goal of this study is to create a collection of enhanced research databases that will add significant socioeconomic and health care access content to the existing datasets hosted on the PDS website, thereby enhancing their analytic capacity and utility. To address existing analytic constraints, the data profiles in selected PDS patient-level cancer phase III clinical datasets have been augmented by linking them with social, economic, and health-related characteristics of similar cancer survivors from nationally representative health and health care-related survey data. This data enhancement project serves to further advance PDS's mission by enabling new explorations into the potential influence of health care access, socioeconomic factors, and health behaviors on the patient-level efficacy and outcomes data contained in the PDS online platform. This data integration effort will help generate collective insights that may yield improvements in trial designs and stimulate new research findings derived from applying advanced analytic methodologies to the content-enhanced datasets.

Using statistical linkage and model-based techniques, patient-level records in selected PDS datasets have been linked to those of comparable cancer survivors, and are thereby augmented with survey content on social, economic, and health-related characteristics. This study provides an overview of the methodologies used to connect patient-level clinical trial data with nationally representative health-related data on cancer survivors from the national Medical Expenditure Panel Survey (MEPS). Research findings include probabilistic assessments of the representation of the patients in the respective clinical trials relative to the characteristics of cancer survivors in the general population. The study also demonstrates how the augmented datasets enable researchers to assess the impact of socioeconomic factors added through data integration on survival and related outcomes of interest.

## Materials and methods

As of April 1, 2018, ProjectDataSphere.org hosts over 145 phase III oncology clinical trial datasets, representing more than 121,000 cancer patients; over the past year, on average 6,200 patients per month were added to this database. The number of datasets continues to expand with uploads from new and existing data providers (3). This data sharing initiative has already demonstrated its benefit to the research community with triple the usage of other major, clinical trial data-sharing efforts combined. PDS data have also been cited by 11 peer-accepted publications on research topics such as the relationship between tumor growth and survival, survival prediction models based on trial design, and meta-analysis of standards of care (4). For selected PDS datasets, our project seeks to extend the utility of these publicly available data by joining PDS patient-level data with nationally representative health-related data from MEPS. MEPS is the nation's primary source of nationally representative, comprehensive, person-level data on health care use, insurance coverage, and expenses. With this additional content, the PDS data platform would further serve to advance cancer research by permitting more granular subgroup and meta-analyses of related treatment protocols. This is particularly important because clinical trials are often conducted among younger, healthier, and less racially diverse patient populations than the population at large (5–7). The augmented datasets should enable researchers to evaluate the efficacy of treatment-vs.-control randomizations and to investigate whether the added variables are related to outcomes of interest. Researchers can also conduct probabilistic assessments of the proportion of the U.S. population that the cancer patient outcomes observed in the PDS online service may or may not represent. The data in the PDS enclave cannot currently support these types of investigations.

The addition of MEPS data to the patient-level data within the PDS enclave will facilitate hypothesis-generating research efforts that explore the level of variation in patient outcomes potentially attributable to differentials in access to basic health care services and their utilization, to socioeconomic characteristics, and to health behaviors and preferences. It will support exploratory analyses designed to examine questions such as:

- Are the demographic characteristics of those cancer patients enrolled in specific phase III clinical trials comparable to cancer patients with the same disease in the general population?
- How are variations in cancer patients' access to health care and income impacting patient outcomes in specific phase III clinical trials?
- What variations in patient outcomes are associated with specific demographic, socioeconomic, and health-related factors?

MEPS is characterized by an integrated survey design. Since its inception, the primary analytical focus of MEPS has been health care access, coverage, cost, and use. Over the past several years, MEPS data have supported a highly visible set of descriptive and behavioral analyses of the U.S. health care system. These include studies of the population's access to, use of, and expenditures and sources of payment for health care; the availability and costs of private health insurance in the employment-related and non-group markets; the population enrolled in public health insurance coverage versus those without health care coverage; and the role of health status in health care use, expenditures, household decision making, and in health insurance and employment choices. Because of the breadth of MEPS data, these data have informed the nation's economic models and their projections of health care expenditures and utilization. The level of cost and coverage detail in MEPS data has enabled public and private sector economic models to develop national and regional estimates of the impact of changes in financing, coverage, and reimbursement policy, as well as estimates of who benefits and who bears the cost of a change in policy.

MEPS has been collecting data on health care utilization and expenditures annually since 1996. The survey is sponsored by the Agency for Healthcare Research and Quality. In addition to collecting nationally representative data to yield annual estimates for a variety of measures related to health care use and expenditures, MEPS provides estimates related to health status, demographic characteristics, employment, health insurance coverage, and access to health care. MEPS consists of a family of three interrelated surveys: Household Component (MEPS-HC), Medical Provider Component (MEPS-MPC), and Insurance Component (MEPS-IC). MEPS-IC also collects establishment-level data on insurance programs. Through a series of interviews with household respondents, MEPS-HC collects detailed information at the level of the individual respondent on demographic characteristics, health status, health insurance, employment, and medical care use and expenditures. These data support estimates both for individuals and for families in the United States. Respondents identify medical providers from whom they have received services (8–10).

The set of households selected for MEPS-HC is a subsample of 15,000 households/35,000 individuals participating in the National Health Interview Survey (NHIS). NHIS is an ongoing annual household survey of ~40,000 households conducted by the National Center for Health Statistics, Centers for Disease Control and Prevention, to obtain national estimates of health care utilization, health conditions, health status, insurance coverage, and access representing the civilian noninstitutionalized population. In addition to the cost savings achieved by eliminating the need to independently list and screen households, selecting a subsample of NHIS participants has led to enhanced analytical capacity of the resultant survey data. Use of NHIS data in concert with the data collected for MEPS provides greater capacity for longitudinal analyses not otherwise available. Furthermore, the large number and dispersion of the primary sampling units in MEPS has resulted in more precise expenditure survey designs. The MEPS-HC survey consists of an overlapping panel design in which any given sample panel is interviewed a total of five times in person over 30 months to yield annual use and expenditure data for 2 calendar years. These rounds of interviewing are conducted at about 5- to 6-month intervals. They are administered through a computer-assisted personal interview mode of data collection, and take place with a family respondent who reports for him/herself and for other family members. Data from two panels are combined to produce estimates for each calendar year.

MEPS-MPC is a survey of the medical providers, facilities, and pharmacies that provided care or services to sample persons. The primary objective is to collect detailed data on the expenditures and sources of payment for the medical services provided to individuals sampled for MEPS. Such data are essential to improve the accuracy of the national medical expenditure estimates derived from MEPS, given that household respondents are not always the most reliable sources of information on medical expenditures. MPC data are collected a year after the household health care event information is collected to allow adequate time for billing transactions to be completed. MPC collects data on dates of visits/services, use of medical care services, charges, sources of payments and amounts, and diagnoses and procedure codes for medical visits/encounters. Only providers for whom a signed permission form was obtained from the household authorizing contact are eligible for data collection in MPC. The categories of providers in MPC include (1) office-based medical doctors; (2) hospital facilities providing inpatient, outpatient, and emergency room care; (3) health maintenance organizations (HMOs); (4) physicians providing care during a hospitalization; (5) home care agencies; and (6) pharmacies. RTI International is the data collection organization for MEPS-MPC.

### Data linkage methodology

The core datasets that are being used for this project consist of historical, patient-level data from academic, and industry phase III cancer clinical trials available on ProjectDataSphere.org and public use files from MEPS. All project members of the team have approved access to the phase III cancer clinical trial data. The MEPS data files are accessible for downloading at the MEPS website (https://meps.ahrq.gov/mepsweb/data_stats/download_data_files.jsp).

The PDS datasets generally represent the comparator arm patients from the related clinical trial. The number of comparator arm patients varies by trial but ranges from roughly 350 to 800 for those trials included in our study. In comparison, the 2013 MEPS public use file, which has a similar sample size to other annual MEPS data files, has more than 2,000 participating sample adults aged 18 and older with a reported cancer diagnosis. This represents multiple types of cancer including more than 225 sample adults with a reported prostate cancer diagnosis, more than 120 sample adults with a reported colon cancer diagnosis, more than 330 sample adults with a reported breast cancer diagnosis, and more than 130 sample adults with a cervical cancer diagnosis, for example. Pooling MEPS cancer survivors across survey years thus results in a much larger set of survivors of a particular cancer type available for linkage.

The statistical linkage between MEPS and PDS data utilizes variables available in both datasets. For most patient-level records on the PDS platform, demographic measures available for statistical linkage are generally limited to age, race, and sex to reduce the possibility of re-identification; a data integration effort limited to these three demographic measures would produce a multitude of many-to-many exact linkages. To ameliorate this problem, our approach to data integration uses an additional measure that further distinguishes patients by their health-related quality of life assessments. This measure is the EQ-5D™ index score, derived from the EuroQoL five dimensions questionnaire, one of the most commonly used measures of health-related quality of life.

The EQ-5D descriptive system consists of the following five health-related components: mobility, self-care, usual activities, pain/discomfort, and anxiety/depression. Each component has three levels for indicating no health problems, moderate health problems, or extreme health problems. A measure for which there are no problems has a level 1 specification, while a component for which there are extreme problems has a level 3 response. Consequently, there are 3^5^ = 243 health states defined by the instrument, with the associated 5-digit response profiles ranging from 11111 for perfect health to 33333 for the worst possible state. To calculate the EQ-5D index score based on the U.S. population-based preference weights, a scoring algorithm has been created and operationalized. For the U.S. general population, the possible EQ-5D index scores range from −0.11 (i.e., 33333) to 1.0 (i.e., 11111) on a scale where 0.0 = death and 1.0 = perfect health (11, 12). The EQ-5D has been administered in past implementations of MEPS, along with the 12-Item Short Form Health Survey (SF-12) developed from the Rand Medical Outcomes Study. The SF-12 is a general health status instrument with 12 questions producing two summary scores, the Physical Component Summary (PCS-12) and the Mental Component Summary (MCS-12). These scores are determined for each adult sample participant in MEPS based on their responses to the SF-12. These respective components are scored such that higher scores represent better physical and emotional function, and are standardized whereby the mean score is 50 and standard deviation is 10 in the general population. Using MEPS responses from the SF-12, predicted values of the EQ-5D index scores can be derived from MEPS for the years the EQ-5D instrument was not administered using an algorithm that only requires the availability of the MCS-12 and PCS-12 scores (13). The prediction model follows:

$$\begin{array}{l} EQ-5D=0.057867+0.010367\;PCS42+0.00822\;MCS42\;\\ \;-0.000034\;PCS42\;MCS42-0.01067 \end{array}$$

Consequently, the statistical linkage process uses a set of discriminatory variables that includes age, race, and sex, and the EQ-5D index score. The EQ-5D score is calculated directly for MEPS years where the EQ-5D questionnaire was administrated and is predicted for years when it was not. When additional demographic measures are available in the PDS data for this statistical linkage (e.g., height, weight, body-mass index, employment status), they are also incorporated in the process. Several years of MEPS data on cancer survivors were pooled to enhance the sample sizes of cases available for linkage for specific cancer classifications. Many-to-many linkages were permitted, which facilitates analysis options where researchers can choose 1-1, many-1, or many-many aggregations. Particular attention is being given to ensuring that the confidentiality provisions of both data sources are satisfied. The approach taken to implement the statistical linkage between the MEPS and select PDS datasets that cover the more prevalent cancers have benefited by related research efforts to optimize the process (14, 15).

MEPS also periodically includes an Experiences with Cancer Survivorship Supplement cosponsored by the Agency for Healthcare Research and Quality, Centers for Disease Control and Prevention, the National Institutes of Health's Office of Behavioral and Social Sciences Research, and LIVESTRONG. The supplement consists of a special set of questions for people who have cancer (16). The MEPS Experiences with Cancer questionnaire asks cancer survivors about the financial costs of cancer, their access to health care, their ability to work and to do normal daily activities, their use of health care and money spent on health care, and their use of prescription drugs. Such content is also available for inclusion in the data augmentation process.

### Linkage of PDS lung cancer patients and MEPS data

To assess the incidence and level of health and health care disparities experienced by cancer patients, much more detailed information beyond the extant measures of age, race, and sex in datasets hosted on PDS is essential to further distinguish their characteristics. Integration with MEPS permits the inclusion of content on demographic characteristics (education level, marital status, family structure); socioeconomic measures (income, poverty status); and health and health care–related measures (health status, number of chronic conditions, access to care, health insurance, medical utilization and expenditures).

To illustrate the data integration enhancements to PDS' analytic capacity, the PDS data file *LungNo_MerckKG_2007*_*145* (https://www.projectdatasphere.org/projectdatasphere/html/content/145) is used as an example. As a general guideline for the reader, for the remainder for the methods section, words that appear capitalized and in parentheses represent the corresponding variable name on the enhanced dataset. The PDS data file includes 507 lung cancer patients, representing the intent-to-treat population. Age, sex, race, and measures of the EQ-5D were used to link to MEPS cases. Each PDS patient completed the EQ-5D questionnaire at multiple points during the study (e.g., at screening, during treatment, at end of study, and possibly multiple times during posttreatment phase), so it was necessary to assign a single health state to each patient prior to linking with the MEPS data. The five dimensions of EQ-5D at baseline were used to derive the EQ-5D summary scores for linkage. Baseline measurements were identified using the PDS variable QSGRPID = “EQ5D – WEEK0.”

MEPS lung cancer survivors were identified among all MEPS cases from the 2000 to 2013 MEPS-HC Survey Full Year Consolidated Data files using the variable ICD9CODX on the Medical Conditions File; it was necessary to link the Full Year Consolidated Data files with the Medical Conditions file to obtain ICD9CODX. MEPS cases with ICD9CODX = 162 were identified as lung cancer survivors.

MEPS lung cancer cases with a non-positive person-level weight were ineligible for inclusion in the linkage process and are not represented in the linked dataset. Table 1 shows the number of MEPS lung cancer cases deemed eligible for linkage; this represents the set of MEPS cases included in the linked dataset. Because MEPS is a panel survey, an individual can be represented in multiple years (maximum of 2 years). Age, sex, race, and measures of the EQ-5D were used to link to PDS cases.

**Table 1:** Number of MEPS lung cancer survivors eligible for linkage by MEPS year.

**Year**	**Count**	**Percent**
2000	28	4.3
2001	37	5.7
2002	49	7.5
2003	46	7.0
2004	46	7.0
2005	36	5.5
2006	33	5.1
2007	49	7.5
2008	60	9.2
2009	53	8.1
2010	61	9.3
2011	59	9.1
2012	49	7.5
2013	47	7.2
Total	653	100.0

*Medical Expenditure Panel Survey Household Component, 2000–2013, Agency for Healthcare Research and Quality, U.S. Department of Health and Human Services*.

### EQ-5D estimation methods

For the PDS data and for MEPS data from 2000 to 2003, responses to the five health measures of the EQ-5D (i.e., mobility, self-care, anxiety/depression, pain/discomfort, and usual activities) were available. Thus, it was possible to directly score a summary value of the EQ-5D (EQ5DDIRECT) using an algorithm developed by Shaw and colleagues in 2005. Additionally, the five measures were used to obtain a predicted value of the EQ-5D (EQ5DDOLAN) based on a modeling approach developed by Dolan (17). For MEPS 2000–2003, the predicted EQ-5D value from the Dolan model was already provided on the source MEPS data files (EQU42). This value was validated, and both the original value from the MEPS data files (EQU42) and the recalculated value from validation (EQ5DDOLAN) are available on the linked PDS-MEPS dataset.

For MEPS 2004–2013 data, only the Physical and Mental Component Summary scores (PCS42, MCS42) from the MEPS Short Form-12 Questionnaire on health status and health care quality were available to calculate a predicted EQ-5D summary score. This prediction method, noted above, was based on the modeling approach developed by Sullivan and Ghushchyan (13).

While matches based on the single-value EQ-5D score are preferable for linkage, categorized values of the EQ-5D were also permitted to allow for tiering of the linkage criteria. The categories were constructed based on the decile classes of predicted EQ-5D values for the PDS cases. For MEPS 2000–2003, where the five measures were available, the predicted EQ-5D values derived from the Dolan model (EQ5DDOLAN) were classified into the decile categories (EQ5DDECILE). For MEPS 2004–2013, where only the MCS and PCS measures were available, the predicted EQ-5D values derived from the Sullivan-Ghushchyan model (EQ5DSG) were classified into the decile categories (EQ5DDECILESG). Note that there are two variables containing the EQ-5D decile categories on the linked dataset (EQ5DDECILE, EQ5DDECILESG), because for MEPS 2000–2003, it was possible to apply the decile criteria to the predicted EQ-5D values from both the Dolan and the Sullivan-Ghushchyan approaches.

Table 2 presents the lower bound of each decile category with the frequency and percent of PDS cases in each class. The top two decile categories were collapsed into a single category (Decile Category = 9), because ~25% of cases had a predicted EQ-5D value of 1. The percentages in each category are not exactly 10% due to ties in the values (i.e., cases with the same EQ-5D value were assigned to the same decile category). Information was missing from 24 PDS cases for the five EQ-5D measures, so a predicted value could not be estimated; these cases are excluded from Table 2.

**Table 2:** Lower bound values for EQ-5D decile categories.

**Decile category**	**Lower bound**	**Count**	**Percent**
1	−0.016	41	8.5
2	0.620	35	7.2
3	0.689	45	9.3
4	0.725	47	9.7
5	0.760	35	7.2
6	0.796	76	15.7
7	0.848	52	10.8
8	0.883	22	4.6
9	1.000	130	27.0

*PDS data file LungNo_MerckKG_2007_145, Project Data Sphere*.

### PDS-MEPS linkage methods

A sequential hierarchical approach was used to link PDS cases to MEPS cases. Each step of the approach represents some degree of relaxation for the linkage criteria, such that linkages obtained at an earlier step are stricter matches than those obtained at a later step. A distinct approach was used for MEPS 2000–2003 versus MEPS 2004–2013, because the available EQ-5D summaries differed between these sets. If either a PDS or MEPS case had a missing value for one for the linkage variables, it did not achieve a linkage.

To link PDS cases with MEPS 2000–2003, the following three-step approach was used.

- In the first step, PDS and MEPS cases were linked when they demonstrated an exact match by single year age, sex, race, and the EQ-5D value directly scored from the five measures. Many-to-many linkages were permitted, so that a PDS case could have been matched with multiple MEPS cases, and MEPS cases may have connected with multiple PDS cases. The resulting set of linkages (i.e., pairs of PDS and MEPS case identification variables) was recorded along with an indicator that the linkages were achieved in the first step of the process.
- In the second step, the full set of PDS and MEPS cases (i.e., both the matched and un-matched cases from the first pass) were linked based on exact matches by categorized age, sex, race, and the EQ-5D value directly scored from the five measures. Age categories included 18–24, 25–34, 35–44, 45–54, 55–64, 65–74, 75–84, and 85+. Again, many-to-many linkages were allowed. Since the linkage criteria used in the first step represent a subspace of the linkage criteria used in the second step, the set of linkages obtained in the first step are a subset of the linkages obtained in the second step. The remaining set of new linkages was recorded with a corresponding indicator describing the second step as the source of these linkages.
- In the third step, the full set of PDS and MEPS cases (i.e., both the matched and un-matched cases from the two prior steps) were linked based on exact matches by collapsed categorized age, sex, race, and the decile categories of the predicted EQ-5D values. Collapsed age categories included 18–24, 25–34, 35–44, 45–54, 55–64, 65–74, 75–84, and 85+. Again, many-to-many linkages were possible. Here, the source of the EQ-5D value used in the third step (i.e., Dolan predicted value based on the five components of the EQ-5D) differed from source in the first two steps (i.e., EQ-5D directly scored from the five components). The remaining set of new linkages was then recorded with a corresponding indicator that the third step was the source of the linkages.

An independent, two-step approach was then used to link PDS with MEPS 2004–2013.

- In the first step, PDS and MEPS cases were linked when they demonstrated an exact match on single year age, sex, race, and the decile categories of the predicted EQ-5D values. Many-to-many linkages were allowed, so that a PDS case may have paired with multiple MEPS cases, and MEPS cases may have paired with multiple PDS cases. The resulting set of linkages from this step (i.e., pairs of PDS and MEPS case identification variables) was recorded along with an indicator that the linkages were achieved in the first step of the approach used for MEPS 2004-2013.
- In the second step, the full set of PDS and MEPS cases (i.e., both matched and un-matched cases from the first step of linking with MEPS 2004-2013) were paired if they demonstrated an exact match on collapsed categorized age, sex, race, and the decile categories of the predicted EQ-5D values. Collapsed age categories included 18–24, 25–34, 35–44, 45–54, 55–64, 65–74, 75–84, and 85+. Again, many-to-many linkages were allowed. Since the linkage criteria used in the first step represent a subspace of the linkage criteria used in the second step, the set of linkages obtained in the first step are a subset of the linkages obtained in the second step. The remaining set of new linkages was then recorded with a corresponding indicator describing the second step as the source of these linkages.

Table 3 summarizes the linkage criteria for each step of the linkage process. The EQ-5D value used for linkage is presented for both MEPS and PDS, since the approach for obtaining predicted EQ-5D values differed for the 2004–2013 MEPS.

**Table 3:** Summary of linkage approach.

**Linkage criteria by step of the sequential approach**	**EQ-5D estimation method**	
	**MEPS**	**PDS**
**2000–2003 MEPS**		
1: Single year age, sex, race, EQ-5D score	Direct	Direct
2: Categorized age, sex, race, EQ-5D score	Direct	Direct
3: Categorized age, sex, race, EQ-5D decile categories	Dolan	Dolan
**2004–2013 MEPS**		
1: Single year age, sex, race, EQ-5D decile categories	Sullivan	Dolan
2: Categorized age, sex, race, EQ-5D decile categories	Sullivan	Dolan

*Medical Expenditure Panel Survey Household Component, 2000–2013, Agency for Healthcare Research and Quality, U.S. Department of Health and Human Services; PDS data file LungNo_MerckKG_2007_145, Project Data Sphere*.

The enhanced database includes the union of the linkages that resulted from the three step process for MEPS 2000–2003 and the two step process for MEPS 2004–2013. Many-to-many linkages were allowed, so a PDS case may have linkages with multiple MEPS cases. Similarly, a MEPS case may have linkages with multiple PDS cases. The variable LINKMETHOD in the enhanced dataset represents the indicator variable created during the linkage process that describes the method, or set of criteria, under which each linkage was attained. This variable is available so that researchers can assess sensitivity of results to the set of MEPS donors used in the analysis.

## Results

### Characteristics of lung cancer cases in PDS clinical trials vs. cancer survivors in the population

Once the PDS cancer survivor data were enhanced via linkage to national health care data from MEPS, the analytical aims of the study could be addressed. A core component of this research effort was to determine how representative the cancer patients enrolled in clinical trials are to like cancer patients in the general population. Consequently, we have focused on examining the sociodemographic and health-related characteristics of those cancer patients enrolled in specific phase III clinical trials relative to the characteristics of individuals in the general population with the same conditions. The MEPS enhanced PDS comparator arm clinical trial data on lung cancer patients, *LungNo_MerckKG_2007_145*, was used to illustrate the capacity of the PDS-MEPS enhanced data to provide insights to these assessments.

As noted above, 653 MEPS lung cancer survivors were identified as candidates for linkage to the 507 PDS lung cancer patients enrolled in the comparator arm of the Merck trial. Using the hierarchical linkage methodology, 401 of the 507 PDS lung cancer patients obtained a linkage with at least one lung cancer survivor represented in the MEPS. Alternatively, 401 of the 653 lung cancer survivors in MEPS achieved at least one linkage to PDS lung cancer cases. This observed differential in linkage rates, conditioned on the characteristics of the cancer patients in the respective datasets, was suggestive of the distinct patient selection criteria that distinguish these trials.

Because the set of lung cancer survivors represented in the pooled MEPS data sets are representative of the lung cancer survivors in the nation, the results of the PDS-MEPS data linkage permitted assessments of the sociodemographic and health-related characteristics that differentiated patients more likely to be represented in the trial. For these analyses, a logistic model was specified to determine the most salient factors that differentiate patients in the PDS trial from their lung cancer survivor counterparts in the overall population. More specifically, lung cancer survivors represented in MEPS with linkage to the patients in the PDS trial were classified as Y = 1, and the unlinked cancer survivors in MEPS were classified as Y = 0. The following sociodemographic and health-related measures were included in the model to determine their significance in distinguishing the likelihood of representation in the PDS lung cancer trial under study (Table 4):

- *Sociodemographic:* Age, race/ethnicity, sex, marital status, employment status, education level, income level, year in MEPS
- *Access related:* Health insurance coverage, ability to obtain necessary medical care
- *Health related:* EQ-5D, perceived health status, limitations in physical functioning, smoker status
- *Health care related:* Office-based physician visits, in-patient hospital stays, emergency room visits, prescription drug purchases, total health care expenditures

**Table 4:** Measures considered as potential predictors of trial linkage status for MEPS lung cancer survivors.

**Measures**	**Description**
Age	Age in years at end of the MEPS survey year
Race	White, Black, Other (including Hispanic)
Sex	Male, Female
EQ-5D decile category	For MEPS 2000–2003, the categorized predicted value of EQ-5D based on Dolan prediction equation. For MEPS 2004–2013, the categorized predicted value of EQ-5D based on Sullivan-Ghushchyan prediction model. Fewer than ten decile categories resulted due to ties. −0.016 ≤ EQ-5D < 0.620 0.620 ≤ EQ-5D < 0.689 0.689 ≤ EQ-5D < 0.725 0.725 ≤ EQ-5D < 0.760 0.760 ≤ EQ-5D < 0.796 0.796 ≤ EQ-5D < 0.848 0.848 ≤ EQ-5D < 0.883 0.883 ≤ EQ-5D < 1.000
	EQ-5D ≥ 1.000
Marital status	Married, Not married (including divorced, separated, widowed, never married)
Employment status	Not employed, Employed at any time during reference period
Education level	No degree, Earned at least GED or high school diploma
Income level	High income (family income ≥400% of the poverty level), poor through middle income (family income < 400% of the poverty level)
MEPS survey period	2000–2003, 2004–2013
Health insurance coverage	Any private insurance, Public insurance only, Uninsured
Smoker status	Current smoker, Not current smoker
Perceived health status	Excellent/Very Good/Good, Fair/Poor
Limitation in physical functioning	Yes, No
Number of prescribed medicine purchases	Frequency in year
Number of hospital discharges	Frequency in year
Number of emergency room visits	Frequency in year
Number of office-based physician visits	Frequency in year
Total health care expenditures	Continuous measure for year
Access to necessary medical care	Able to get access, Unable to get access

*Medical Expenditure Panel Survey Household Component Data Files 2000–2013, Agency for Healthcare Research and Quality, U.S. Department of Health and Human Services*.

Our multivariate logistic regression analyses helped identify the set of significant factors (*p* < 0.05) that were more characteristic of the PDS lung cancer patients enrolled in the trial relative to adult lung cancer survivors in the U.S. noninstitutionalized population (Table 5). Based on the results of the logistic model, the following measures were identified as significant predictors (*p* < 0.05) of having a greater likelihood of being represented in the trial: race/ethnicity, sex, marital status, MEPS survey year, EQ-5D, and smoker status. More specifically, the lung cancer patients enrolled in the trial were more likely to be men, white, married, and current smokers relative to their representation in the population. Individuals characterized by fewer health problems as noted by higher values of the EQ-5D were also more likely to be enrolled in the trial.

**Table 5:** Logistic regression model to identify factors associated with trial linkage status for MEPS lung cancer survivors.

**Independent variables and effects**	**Beta coeff**.	**SE beta**	***p-*value *t*-test B = 0**	**d.f**.	**Wald F**	***p*-value Wald F**
Overall model				9	6.95	< 0.0001
Intercept	1.06	0.43	0.0145			
Marital status				1	6.16	0.0134
Married	1.01	0.40	0.0134			
Sex				1	11.04	0.0010
Female	−1.61	0.49	0.0010			
MEPS Survey Year				1	6.47	0.0113
2000–2003	−0.93	0.37	0.0113			
EQ-5D Decile Category	0.26	0.07	0.0001	1	14.81	0.0001
Race				2	25.94	< 0.0001
Other (including Hispanic)	−4.39	0.85	< 0.0001			
Black	−5.93	0.91	< .0001			
Access to necessary medical care				1	3.17	0.0758
Unable to get access	1.09	0.61	0.0758			
Smoking status				1	4.46	0.0352
Current smoker	0.94	0.44	0.0352			

*n = 470*.

*Analysis performed using SUDAAN statistical software. The subpopn statement was used to conduct the subpopulation analysis of lung cancer survivors from among all MEPS cases (2000–2013)*.

Pseudo R-square: 0.429977

−2 ^*^ Normalized log-likelihood with intercepts only: 580.32

−2 ^*^ Normalized log-likelihood full model: 316.14

Approximate chi-square (−2 ^*^ log-L ratio): 264.18

Degrees of freedom: 8

Denominator degrees of freedom: 445

*Medical Expenditure Panel Survey Household Component Data Files 2000–2013, Agency for Healthcare Research and Quality, U.S. Department of Health and Human Services*.

The inclusion of the MEPS survey year variable was a methodological consideration, serving to control for the estimation strategy utilized for the EQ-5D measurement. In these analyses, the standard errors of the survey estimates and model coefficients derived from MEPS have been adjusted for the impact of clustering due to the multistage survey design, and the test statistics used to test for equivalence in estimates and significance in model coefficients have also been adjusted to control for survey design complexities. As a consequence of these data integration efforts to enhance the analytic utility of the PDS clinical trial data content, comparable studies assessing the representation of other clinical trial data hosted on the PDS website can be undertaken.

### Informing health disparities research

Studies assessing the presence of health disparities in the care provided to cancer patients have benefitted from the availability of sociodemographic and national health care data. National health data have been used to understand the relationship between insurance coverage and level of care received by cancer patients. In a study of cancer survivors from 2011 MEPS, patients who reported barriers to necessary cancer care were more likely to represent those with lower educational attainment and those having no insurance or public insurance (18). Additionally, data from the 2012 to 2015 National Health Interview Survey (NHIS) were used to examine the impact of policy changes resulting from implementation of the Affordable Care Act (ACA) on insurance coverage for nonelderly adult cancer survivors compared to adults who did not have a history of cancer; this study found that the rate of uninsured cancer survivors declined with implementation of the ACA and that cancer survivors who were eligible for Medicaid experienced greater coverage gains from the ACA than the adults without cancer (19). The NHIS was also used to explore longitudinal trends of cost-related medication nonadherence among a national sample of U.S. cancer survivors, which demonstrated a significant increasing trend among younger cancer survivors after controlling for demographic and socioeconomic factors (20). Disparities related to several demographic, health insurance coverage and health care access factors have been observed in the utilization of screening tests for breast, cervical, and colorectal cancer based on analysis of the NHIS data (21).

National data have also provided insights into the impact of socioeconomic status and race/ethnicity on the quality of care received. The MEPS Experiences with Cancer Supplement was recently utilized to determine whether racial/ethnic disparities exist in the quality of patient-provider communication during treatment among breast cancer patients (16). Study findings revealed that, when controlling for factors such as income and health insurance coverage, the quality of patient-provider communication with breast cancer patients varies by race/ethnicity with non-Hispanic blacks experiencing the greatest communication deficit (22). Another population-based analysis of National Cancer Data Base records for invasive primary epithelial ovarian cancer revealed significant differences in adherence to National Comprehensive Cancer Network (NCCN) guidelines for care and overall survival according to measures of race and socioeconomic status (23). These data highlight statistically and clinically significant disparities in the quality of ovarian cancer care and overall survival, independent of NCCN guidelines, along racial and socioeconomic parameters.

In another study, Behavioral Risk Factor Surveillance System data were utilized to determine the extent of racial/ethnic disparities in colorectal cancer screening (CRC) in the nation (24). Study findings indicated the presence of large racial/ethnic disparities in CRC screening, with substantial differences for minorities that were still present when controlling for socioeconomic factors and access to care. Disparities in cancer incidence and mortality have also been informed utilizing cancer incidence data from the National Cancer Institute, the Centers for Disease Control and Prevention, and the North American Association of Central Cancer Registries, and mortality data from the National Center for Health Statistics. According to the American Cancer Society, as of 2016, while blacks were observed to continue to experience higher cancer death rates than whites, the disparity has narrowed for all cancers combined in men and women and for lung and prostate cancers in men. Furthermore, the racial gap in death rates was observed to widen for breast cancer in women but did not vary for colorectal cancer in men (25).

While the data available in the PDS website are rich in measures that characterize the clinical trials under study, the treatment protocols, and patient outcomes, the data providers significantly limit the inclusion of many demographic, socioeconomic, and health care-related measures. Prior to this initiative, the influence of health-related and socioeconomic factors, access to and use of health care services, and predisposition of health behaviors on treatment effects and patient outcomes could not be assessed with PDS data. For example, an analysis to determine the set of factors associated with survival for the lung cancer patients in the comparator arm of the PDS clinical trial was dependent on the restricted set of clinical measures made available by the data provider. These included patient demographics, EQ-5D measurement, performance on the Eastern Cooperative Oncology Group (ECOG) scale, response to chemo-radiotherapy, type of chemo-radiotherapy, tumor cancer stage, histology, and smoking history (Table 6). Alternatively, with the inclusion of the linked MEPS lung cancer survivor patient data, additional content on person-level characteristics, preferences, access to care, insurance coverage, health and health care-related measures are now available to enhance research efforts (Table 7).

**Table 6:** Measures from PDS considered as potential predictors of survival status for PDS lung cancer patients.

**Measures**	**Description**
Age	Age in years
Race	White, Other
Sex	Male, Female
Most Recent EQ-5D Measurement	Most recently recorded EQ-5D measurement
ECOG Performance	Scale used to assess how a patient's disease is progressing and how the disease affects daily living abilities:
	Fully active without any physical restriction
	Restricted in physical activity of a strenuous nature
Response to Chemo-radiotherapy	Partial/complete response, stable disease
Type of Chemo-radiotherapy	Concomitant, Sequential
N Stage	Cancer stage that describes the number and relative location of lymph nodes affected by the tumor. A higher number after the N indicates that a greater number of lymph nodes have been affected:
	NX/N0 (Not measurable; no cancer)
	N1/N2
	N3
Histology	Adenocarcinoma, Squamous cell carcinoma, Other/Unknown
Smoking History	Active smoker, Nonsmoker/former smoker

*Data files from LungNo_MerckKG_2007_145 accessed via Project Data Sphere*.

*Unless otherwise noted, the variables represent measurements taken at baseline or screening*.

**Table 7:** Measures from MEPS considered as potential predictors of survival status for PDS lung cancer patients.

**Measures**	**Description**
Marital status	Married, Not married (including divorced, separated, widowed, never married)
Employment status	Not employed, Employed at any time during reference period
Education level	No degree, Earned at least GED or high school diploma
Income level	High income (family income ≥400% of the poverty level), poor through middle income (family income < 400% of the poverty level)
Private insurance coverage	Yes, No (including public insurance only or uninsured)
Smoker status	Current smoker, Not current smoker
Belief: Health insurance not needed	Disagree/Uncertain, Agree
Belief: Health insurance not worth cost	Disagree/Uncertain, Agree
Belief: More likely to take risks	Disagree/Uncertain, Agree
Belief: Able to overcome illness without help	Disagree/Uncertain, Agree
Perceived health status	Excellent/Very Good/Good, Fair/Poor
Limitation in physical functioning	Yes, No
Number of prescribed medicine purchases	Frequency in year
Number of hospital discharges	Frequency in year
Number of emergency room visits	Frequency in year
Number of office-based physician visits	Frequency in year
Total health care expenditures	Continuous measure for year
Access to necessary medical care	Able to get access, Unable to get access
Medicare coverage	Covered, Not covered
Medicaid coverage	Covered, Not covered
Tricare coverage	Covered, Not covered
Private HMO coverage	Covered, Not covered
Office-based provider visit: EEG^*^	Yes, No (including no provider visits)
Office-based provider visit: EKG^*^	Yes, No (including no provider visits)
Office-based provider visit: MRI^*^	Yes, No (including no provider visits)
Office-based provider visit: lab tests^*^	Yes, No (including no provider visits)
Office-based provider visit: anesthesia^*^	Yes, No (including no provider visits)
Office-based provider visit: other exams^*^	Yes, No (including no provider visits)

*Medical Expenditure Panel Survey Household Component Data Files 2000–2013*,

* *Medical Expenditure Panel Survey Office-Based Medical Provider Visits Files 2000–2013. Agency for Healthcare Research and Quality, U.S. Department of Health and Human Services*.

To illustrate the gains in analytic capacity achieved through data integration when conducting survival analyses, we examined the significance of the inclusion of the set of the additional health-related predispositional measures obtained from MEPS to discern suggestive relationships with mortality. A logistic regression analysis was conducted on PDS-MEPS enhanced data on lung cancer patients to identify factors associated with survival status for PDS lung cancer patients. The results indicated that, in addition to the PDS measure reflecting the stage of the lung cancer tumor, a cancer patient's likelihood of survival was associated with their insurance coverage status, their status as a smoker, their health preferences, and the intensity of services received in their ambulatory health care visits (Table 8). Based on the enhanced data, lung cancer patients in the comparator arm of the trial who had Medicaid coverage (*P* = 0.005) or private HMO coverage (*P* < 0.10) were characterized by a greater likelihood of survival than their counterparts. Service-intensive office-based health care visits that included lab tests were also associated with a greater likelihood of survival (*P* < 0.01). Alternatively, lung cancer patients characterized as current smokers (*P* < 0.10) were associated with a lower likelihood of survival. Lung cancer patients tied to a belief that health insurance is not needed, suggestive of a more self-reliant persona, were also associated with a greater likelihood of survival (*P* = 0.03).

**Table 8:** Logistic regression model to identify factors associated with survival status for PDS lung cancer patients.

**Independent variables and effects**	**Beta coeff**.	**SE beta**	***p*-value *t*-test B = 0**	**d.f**.	**Wald F**	***p*-value Wald F**
Overall model				8	9.63	<0.0001
Intercept	0.37	0.51	0.4748			
N Stage				2	4.12	0.0170
N1/N2	−0.42	0.50	0.4041			
N3	0.54	0.57	0.3379			
Medicaid coverage				1	8.07	0.0048
Covered	1.61	0.57	0.0048			
Private HMO coverage				1	3.76	0.0531
Covered	0.59	0.31	0.0531			
Smoking status				1	2.77	0.0969
Current smoker	−0.49	0.29	0.0969			
Belief: Health insurance not needed				1	4.86	0.0282
Agree	1.78	0.81	0.0282			
Office-based visit: lab tests				1	7.38	0.0069
Yes	0.80	0.29	0.0069			

n = 356 Pseudo R-square: 0.092231

−2 ^*^ Normalized log-likelihood with intercepts only: 424.65

−2 ^*^ Normalized log-likelihood full model: 390.20

Approximate chi-square (−2 ^*^ log-L ratio): 34.45

Degrees of freedom: 7

Denominator degrees of freedom: 355

*Data files from LungNo_MerckKG_2007_145 accessed via Project Data Sphere. Medical Expenditure Panel Survey Household Component Data Files 2000–2013, Medical Expenditure Panel Survey Office-Based Medical Provider Visits Files 2000–2013, Agency for Healthcare Research and Quality, U.S. Department of Health and Human Services*.

## Discussion

PDS is a platform that provides the research community with broad access to both de-identified patient-level data from oncology clinical trials and related analytic tools after completing a brief application, with no research merit review required to access the data. There are currently more than 1,850 authorized users with access to over 145 datasets on the PDS platform, representing over 120,000 patient lives and a broad array of tumor types. Site activity has increased significantly since PDS was launched in April 2014; over 10,000 data downloads for research purposes have occurred since that time. The achievement of these research milestones was made possible through collaborations with organizations that provided data and catalyzed the use of the platform for research innovation. Charter data providers include AstraZeneca, Bayer, Celgene, Janssen, Memorial Sloan Kettering Cancer Center, Pfizer, and Sanofi. Most of these organizations added data following launch. Additionally, the ranks of data providers grew to include other leading oncology research organizations such as Amgen, Clovis, EMD Serono, Lilly, Millennium, Synta; and the Alliance for Clinical Trials in Oncology and ECOG-ACRIN, two of the five U.S. network groups of the National Cancer Institute's National Clinical Trials Network Program. Additional National Cancer Institute data are made available through a link with the NCTN/NCORP Data Archive. The 1,850 PDS authorized users, its data providers and supporters, and others in its external network are being updated on the project's scope, progress, new enhanced analytic datasets, and related research findings via website and e-mail outreach.

While the data provided to PDS are rich in measures that characterize the clinical trials under study, data providers are required to de-identify patient-level data by removing key demographic data. To assess the incidence and level of health and health care disparities experienced by cancer patients, much more detailed information will need to be added to the PDS datasets, beyond the extant measures of age, race, and sex, to further distinguish their characteristics. To address these analytic constraints, the data profiles in selected PDS patient-level cancer phase III clinical datasets have been augmented by linking the social, economic, and health-related characteristics of like cancer survivors from nationally representative health and health care-related survey data. More specifically, PDS and RTI collaborated on a project to permit the use of socioeconomic, access, and health care-related data to enhance the analytical utility of selected datasets (www.ProjectDataSphere.org). This initiative was undertaken to enable the PDS user community to investigate a broader array of research questions regarding factors that may impact patient outcomes, and to inform studies on identifying health-related disparities. In addition to the clinical trial data on lung cancer patients highlighted in the prior sections, several other enhanced datasets are now available to all authorized PDS platform users. Of these, several PDS datasets have been supplemented with socioeconomic and health care content representing prostate and lung cancer and multiple myeloma.

Using data integration methods, this study linked sociodemographic, access, health, and health care-related measures associated with a nationally representative set of lung cancer survivors included in MEPS to similar cancer patients in the PDS analytic datasets. In addition to utilizing demographic information (age, race/ethnicity, and sex) available in both data sources, the data integration was further advanced by including responses to patient-reported outcomes data captured in the EQ-5D index score derived from the EuroQoL five-dimensions questionnaire. The data integration with MEPS now facilitates the inclusion of content on demographic characteristics (education level, marital status, family structure); socioeconomic measures (income, poverty status); and health and health care-related measures (health status, number of chronic conditions, access to care, health insurance, medical utilization, and expenditures).

The measures appended to each patient-level record in selected datasets hosted on PDS are in the form of data vectors or distributions derived from MEPS, including a weight that supports population-level inference. Comparison of these data vectors and these families of distributions will help enable researchers to investigate whether the added measures potentially affect cancer patient outcomes. More specifically, this collaboration has produced the following analytic enhancements:

- a collection of content-enhanced PDS datasets for patients with the more prevalent cancers, achieved by linking the social, economic, and health-related characteristics of like cancer survivors from nationally representative health and health care–related survey data;
- a capacity to evaluate the alignment of the predispositional characteristics of patients in the study comparator arms for selected datasets and assessment of the level of representativeness in the population of the cancer patients in the respective trials;
- a capacity to assess the reproducibility of analytic findings obtained from the enhanced and integrated PDS datasets;
- dissemination of the set of enhanced analytic datasets to researchers through the PDS online service, facilitating enhanced analyses that explore levels of variation in treatment effects and patient outcomes potentially attributable to differentials in access to health care and more detailed socioeconomic characteristics; and
- dissemination of the methodology employed to allow researchers to achieve comparable analytic enhancements to existing PDS datasets by implementing this methodology for integrating essential additional data on cancer survivors from nationally representative health and health care-related survey data.

The PDS *LungNo_MerckKG_2007_145* comparator arm clinical trial data on lung cancer patients demonstrated the methodology employed to link national health care survey content, thereby enhancing analytic capacity. An analysis of the PDS-MEPS enhanced data to provide insights to these assessments helped to discern the characteristics of the lung cancer patients enrolled in the trial relative to all adult lung cancer survivors in the U.S. noninstitutionalized population. The results indicated that the lung cancer patients enrolled in the trial were more likely to be men, white, married, smokers, and in better health relative to their counterparts represented in the population at large. These findings align with other assessments of study representativeness that indicate that clinical trials are often conducted among younger, healthier, and less racially diverse patient populations than the population at large.

Health disparities for individuals with cancer are most apparent when there are notable differences in the occurrence, frequency, death, and burden of cancer among specific population groups, which often are manifest when comparing the experiences of distinct racial and ethnic minority groups. Poverty, lack of access to prevention/detection services, and the unavailability of high-quality treatment are factors that influence such differentials in patient outcomes. Consequently, research efforts that focus on the determinants of health disparities depend on the availability of information that distinguish cancer patients by demographic and socioeconomic factors, their access to health care services and treatments, and their health behaviors. With the inclusion of linked MEPS lung cancer survivor patient data added to the PDS trial data, this additional content on person-level characteristics, health care preferences, access to care, insurance coverage, health and health care-related measures now permits exploratory studies to identify extant health-related disparities. For each MEPS enhanced dataset on the PDS website, supporting documents have been provided that contain detailed information on this enhanced data content and the data linkage methodology. Researchers can now access the data and supporting documents by logging in and following the links on the RTI International–Project Data Sphere, LLC, Collaboration overview page https://www.projectdatasphere.org/projectdatasphere/html/landing/rti. As additional clinical trial datasets are added to the PDS website, researchers can also initiate future data augmentations using MEPS by implementing the delineated linkage methodology.

## Author contributions

SC contributed to the conception and design of the study and wrote the first draft of the manuscript; SC and JU organized the database, performed the statistical analysis, and wrote sections of the manuscript. All authors contributed to manuscript revision, and read and approved the submitted version.

### Conflict of interest statement

The authors declare that the research was conducted in the absence of any commercial or financial relationships that could be construed as a potential conflict of interest.
